# Piloting online self-audit of methadone treatment in Irish general practice: results, reflections and educational outcomes

**DOI:** 10.1186/s12909-018-1259-2

**Published:** 2018-06-27

**Authors:** Marie Claire Van Hout, Des Crowley, Aoife McBride, Ide Delargy

**Affiliations:** 1 Public Health Policy and Practice, Public Health Institute, Liverpool John Moore’s University, Liverpool, UK; 2 Substance Misuse Programme, Irish College of General Practitioners, Dublin, Ireland; 3 Irish College of General Practitioners, Dublin, Ireland

**Keywords:** Audit, Self-audit, Methadone, Reflective practice

## Abstract

**Background:**

Work based learning underpins the training and CPD of medical practitioners. Medical audit operates on two levels; individual self-assessment and professional/practice development. In Ireland, annual practice improvement audit is an essential requirement for the successful completion of continuous professional development (CPD) as determined by the regulatory body, the Irish Medical Council. All general practice (GP) doctors providing methadone maintenance treatment (MMT) in Ireland have a contractual obligation to partake in a yearly methadone practice audit. The Irish College of General Practitioners (ICGP) as national training provider is tasked to facilitate this annual audit process. The purpose of this audit is to assess the quality of care provided to patients against an agreed set of national standards, enhance learning, and promote practice improvement and reflective practice. The aim was to present an online MTP self-audit and evaluate results from a 12-month pilot among GPs providing MMT in Ireland.

**Methods:**

A mixed method study describing three phases (design and development, pilot/implementation and evaluation) of a new online self –audit tool was conducted. Descriptive and thematic analysis of audit and evaluation data was conducted.

**Results:**

*Survey Monkey* is a suitable software package for the development and hosting of an easy to use online audit for MMT providing doctors. Analysis of the audit results found that the majority of GPs scored 80% or over for the 25 identified essential criteria for MMT provision. The evaluation of the GP audit experience underscores the positive outcomes of the online self-audit in terms of improving practice systems, encouraging reflective practice, enhanced patient care and doctor commitment to continued provision of MMT in addiction clinics and in primary care.

**Conclusions:**

Results from this audit demonstrate a high level of compliance with best practise MMT guidelines by Irish GPs providing MMT. The online self-audit process was well received and encouraged reflective practice. The audit process hinged on the individual GP’s ability to review and critically analyse their professional practice, and manage change. This model of audit could be adapted and used to monitor the management of other chronic illnesses in general practice.

## Background

Work based learning underpins the training and continuing professional development (CPD) of medical practitioners [1–3]. Medical audit forms part of CPD and involves the assurance, monitoring, and improving of quality of professional standards [4, 5]. Audit operates on two levels: individual self-assessment and professional development and the clinical review of team performance contributing to enhanced quality of systems and operation [6]. Frequent written and verbal formative feedback using criterion referenced practice audits encourage critical self-assessment and reflection around patient care and unique practice operations [7–11]. Cochrane reviews have demonstrated how audit can cause small to moderate effects in improving professional practice [12]. Successful audit processes hinge on audit feedback being most effective when provided concurrently within an audit cycle [13, 14]. The focus centres on supported participation in practice, encouragement of self-directed learning [15, 16], and reflective and reflexive practice [17].

The topic of interest for this Irish study centred on the development of an audit process to assess the quality of care provided by general practitioners (GPs) to opiate dependent patients on methadone maintenance treatment (MMT) so as to ensure that patient care meets national and international best practice standards, and also to enhance practice based learning and reflective practice. Ireland has developed a primary care model for delivering MMT for opioid dependent patients, with prescribing GPs generally positive in attitude toward MMT [18]. Opiate dependence is characterised as a chronic, relapsing disorder with permanent metabolic deficiency [19], and is complex in terms of successful treatment, which generally requires long term pharmacological (for example methadone and buprenorphine), psychosocial and relapse prevention treatment modalities [20]. The provision of MMT in Irish primary care is guided by the Methadone Treatment Protocol (MTP) which provides systematic protocols for prescribing, patient management, GP education and clinical auditing as quality assurance and educational tools. MMT is initiated by specialist trained Level 2 GPs or in addiction clinics and once stabilised, patients’ treatment can be moved to Level 1 GPs, with less specialist training, working in primary care.

The Irish College of General Practitioners (ICGP) is the national postgraduate and CPD provider for all GPs and provides training and education to both level 1 and level 2 GPs involved in MMT provision. The ICGP Substance Misuse Programme (SMP) team provide support and mentoring to GPs experiencing difficulties in achieving MTP standards. Ultimately the audit is intended to improve patient care and safety, minimise methadone diversion, reduce drug overdose rates, address associated health conditions and optimise patient rehabilitative outcomes within the practice. The ICGP facilitates quality assurance of MMT via the MTP in Ireland using a newly designed blended method of online self and external audit. The first pilot phase of the online MTP self-audit was undertaken in 2016. The GPs ability to review and critically analyse their professional practice according to the MTP standards, along with the management of professional and practice learning and change is encouraged within the blended audit cycle [21]. We present here the online self-audit development, pilot/implementation and evaluation process outcomes for the first 12-month MTP self-audit cycle for GPs providing MMT in Ireland.

## Methods

### Phase one: Development (descriptive)

**Fig. 1:**
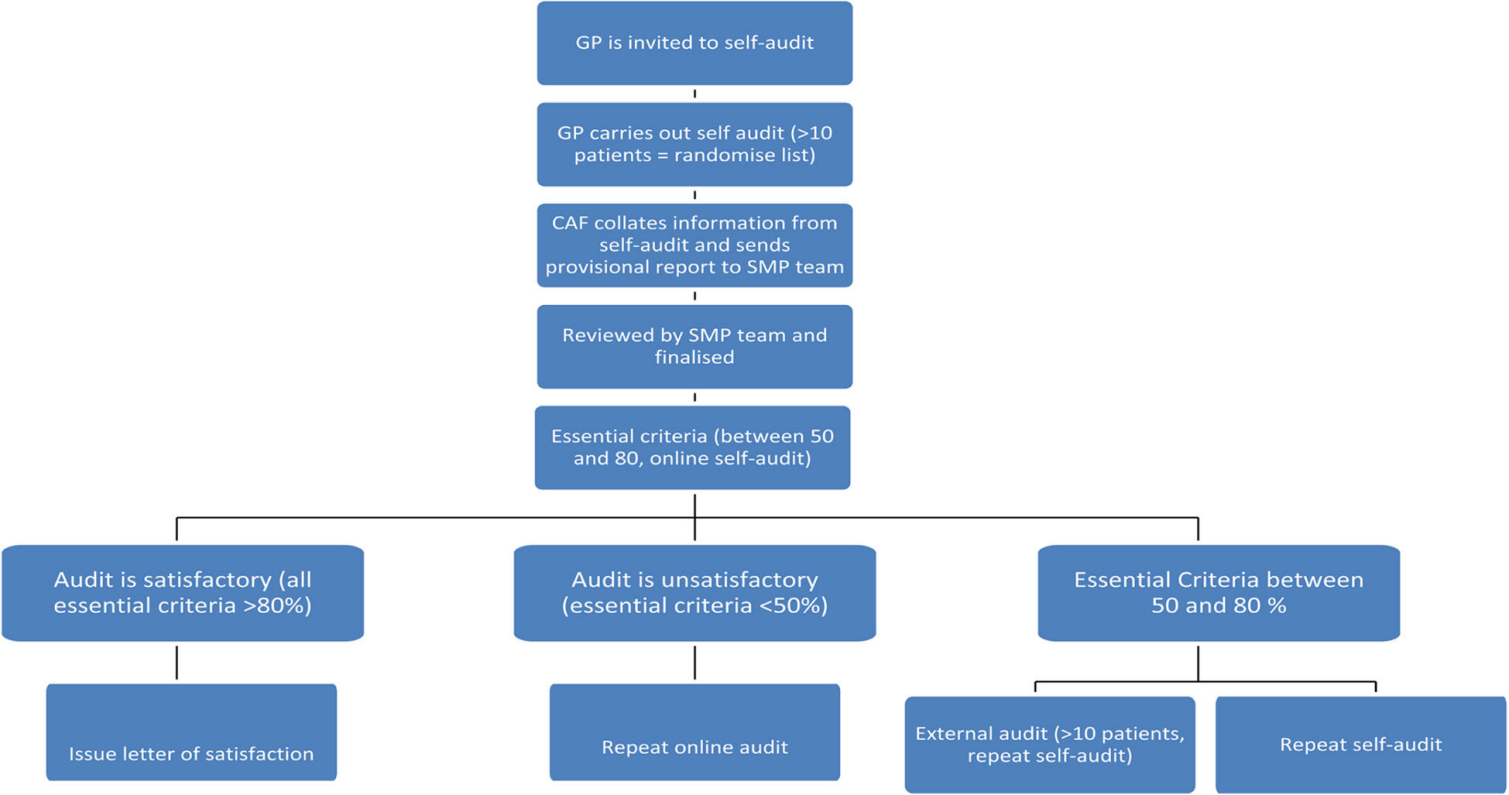
The MTP Audit Cycle

All documentation at the ICGP related to the development of the online audit tool was retrieved and reviewed by the SMP research team. The retrieved data was organised chronologically to understand the development process. Author two drafted the processes undertaken in the development of the tool and this was reviewed and checked for accuracy by authors one and three (Fig. 1).

### Phase two: Implementation (quantitative)

In 2016, all Level 1 and 2 GPs in the national sample (*n* = 345) received the online self-audit tool based on the MTP standards (Table 1), were asked to select a four-week period and complete a chart review on a randomised list of 10 methadone patients in their practise. Informed and written consent was given by all participants prior to participation. If the GP had fewer than 10 patients they were required to undertake the audit on all of their patients. 182 individuals completed the online self-audit. Anonymised results from the pilot phase (12 months) were collated using *Survey Monkey* and descriptive analysis including frequencies and percentages was undertaken using SPSS 10.

**Table 1:** The MTP Audit Criteria

PATIENT INFORMATION/CONSENT	
	Criteria
1.1	It is documented that the patient be advised of the audit process
PATIENT HISTORY	
	Criteria
2.1.1	Patients transferred to the practice for continuation of methadone treatment have a documented completed transfer summary/initial assessment
2.1.2^a^	Patients who commence treatment in the practice have a documented initial assessment
2.1.3^a^	Patients commencing treatment in the practice have a record of 3 drug screens confirming findings from assessment
2.1.4	One of the 3 drug screens prior to commencing treatment is a 6 am test
2.1.5^a^	Patients have been given information on methadone safety, risks and safe storage
MONITORING METHADONE TREATMENT	
	Criteria
3.1.1^a^	All consultations with the GP are recorded
3.2.1^a^	Patients have current dose of methadone recorded at each visit
3.2.2	The rational for the dose of methadone if outside the therapeutic range is recorded
3.3.1^a^	Patients have frequency of supervised dispensing recorded
3.3.2	Patients have rationale for supervised dispensing recorded
3.4.1	Is there a record of a minimum of one randomised drug screen per month?
3.4.2	All drug screens are positive for methadone/EDDP
3.5.1^a^	There is a record if patients are using other substances along with their methadone.
3.5.2^a^	There is evidence of the interventions made if patients are using other substances along with their methadone (opiates, benzodiazepines, cocaine, alcohol, cannabis etc.)
PRESCRIBING OF BENZODIAZEPINE AND Z-HYPNOTIC DRUGS	
	Criteria All patients who are prescribed Benzodiazepines should have recorded in their notes:
3.6.1^a^	The rationale for prescribing
3.6.2^a^	A review of the Rx in the last 3 months
3.6.3^a^	The dose
3.6.4^a^	Evidence of 3 monthly reviews
3.6.5^a^	Evidence of communication with prescriber (if GP is not main prescriber)
	Criteria All patients who are prescribed Z-Hypnotics should have recorded in their notes:
3.7.1^a^	The rationale for prescribing
3.7.2^a^	A review of the Rx in the last 3 months
3.7.3^a^	The dose
3.7.4^a^	Evidence of 3 monthly reviews
3.7.5^a^	Evidence of communication with prescriber (if GP is not main prescriber)
VIRAL SCREENING, AFTERCARE AND IMMUNISATION	
	Criteria
4.1.1^a^	There is a record that patients at risk of blood borne viruses have been offered screening for HIV
4.1.2^a^	There is a record that patients at risk of blood borne viruses have been offered screening for Hepatitis C
4.1.3^a^	There is a record that patients at risk of blood borne viruses have been offered screening for Hepatitis B
4.1.4	There is a record that patients at risk of blood borne viruses have been offered screening for Hepatitis A
4.2.1^a^	There is evidence that patients who are HIV positive have been referred to specialist service.
4.2.2^a^	There is evidence that patients who are Hepatitis C antibody positive and PCR positive/ PCR testing not available have been referred to specialist service as appropriate.
4.3.1^a^	There is evidence that patients who are Hep B negative have been offered vaccination.
4.3.2	All patients who have completed Hep B vaccination regime have been offered post vaccination Hep B antibody test
4.3.3	There is evidence that patients who are Hep A antibody negative have been offered Hep A vaccination

^a^ *denotes essential criteria*

### Phase three: Evaluation (qualitative)

An online open-ended survey investigating GPs perspectives on the process and the complexities of learning in methadone practice was administered to all individuals who had completed the audit and had received their result were emailed an invitation to complete the online evaluation tool (*n* = 132). Informed and written consent was given by all participants prior to participation. Participants gave permission for the use of quotes retrieved from the open ended survey questions. After one month, a reminder was emailed, with 57 individuals completing the evaluation. Descriptive statistical and content analysis was undertaken with assistance from SPSS/QSR NVivo respectively [22]. Content analysis involved open, axial and selective coding of open ended text data which resulted in the generation of listing of key concepts, ideas, words and phrases, formulating main and sub categories, and generating overarching themes.

## Results

The results are reported separately for each phase of the study.

### Phase one: Development (descriptive)

Prior to the development of this on-line audit tool, previous MTP audits were either an external audit conducted by the clinical audit facilitator as part of a practice visit (GPs with more than five patients) or a paper audit (GPs with five or less patients). The process was considered inefficient, with only small numbers of those requiring audit completing it annually. The original audit template consisted of a series of criteria based on national and international opioid treatment guidelines. This template included criteria on consent for audit, assessment of dependence, monitoring of treatment, documentation and review of MMT, the prescribing and monitoring of benzodiazepines and other psychoactive drugs, and the screening and management of blood borne viruses (BBVs). A number of computer platforms were considered. Many were deemed unsuitable due to cost and the need for computer expertise in the ongoing development of the tool. *Survey Monkey* was chosen as the most suitable platform. The platform allowed the development team to create an online audit tool that could easily be adapted by the onsite team with basic computer skills. It allowed modifications (following numerous pilots) to be made with ease and without the need to source external expensive expertise. The tool had the ability to analyse the data inputted which could easily be compared with the expected standard for each criterion. This could easily be retrieved in electronic form and sent to the GP with recommendations on practise improvement. Once the platform was agreed, the original audit criteria were updated using more up to date guidelines and adapted for use in the *Survey Monkey* format. (See Table 1).

Criteria were defined as essential or recommended based on the quality of the evidence available. An Audit Review Group (ARG) at the ICGP provided governance for the project and decided on the required standard for each of the essential criteria. Different audit cycles were developed depending on the standards achieved and included a successful outcome or a repeat audit using a mix of external and /or repeat self-audit (see Table 2).

**Table 2:** The MTP Audit Standards

Overall standard between 80 and 100%: The audit is satisfactory and the GP may continue with self-audit.	
Overall standard between 50 and 80%: The audit result will be reviewed by the Audit Review Group after which the CAF will contact the GP to discuss the recommendations made by the ARG, and to offer the GP additional support with their next audit. Depending on the number of recommendations their next audit may be an external or online self-audit.	
Overall standard below 50% and any or all of the criteria - The audit will be reviewed by the Audit Review Group and the CAF will contact the GP to discuss the recommendations made by the Audit Review Group. The CAF and the local GP Coordinator are available to support the GP to implement these recommendations. Once these are completed the GP will undergo an external audit. Successful completion of this audit will enable the GP to return to the self –audit cycle.	
If the overall standard remains below 50% following this external audit, the Audit Review Group will liaise with the relevant health service manager with a view to identifying supports to improve the quality of outcomes.	

### Phase two: Implementation (quantitative)

Over half (*n* = 182) (53%) of all GPs providing MMT completed the on-line survey. The majority of participants achieved the agreed standard (≥ 80%) particularly for the essential criteria, which ranged from 66.9 - 100%. Over 90% of GPs attained the agreed standard in 14 of the 25 essential criteria and over 80% achieved the required standard in 21 of the 25 essential criteria. The least successful essential criteria were those related to communication with other prescribers of benzodiazepines (56.9%) and the offering of Hepatitis B vaccination to patients who were found to be negative for Hepatitis B Virus (HBV) on screening (66.9%). The non-essential criteria causing the most difficulty for GPs were those related to the provision of information on consent for audit (39.6%) and the offering of Hepatitis A screening (44.2%) and vaccinations (43.5%) (Table 3).

**Table 3:** Implementation Results

	Criterion:	Responses (n)	Achieved required Standard ≥80% n (%)
1.1	It is documented that patient be advised of the audit process	182	72 (39.6)
2.1.1^a^	Patients transferred to the practice for continuation of methadone treatment have a documented completed transfer summary/initial assessment	174	136 (78.2)
2.1.2^a^	Patients who commence treatment in the practice have a documented initial assessment	70	62 (88.6)
2.1.3^a^	Patients commencing treatment in the practice have a record of 3 drug screens confirming findings from assessment	69	66 (95.7)
2.1.4	1 of the 3 drug screens prior to commencing treatment is a 6 am test	53	51 (96.2)
2.1.5^a^	Patients have been given information on methadone safety, risks and safe storage	181	153 (84.5)
3.1.1^a^	All consultations with the GP are recorded	178	178 (100)
3.2.1^a^	Patients have current dose of methadone recorded at each visit	182	175 (96.2)
3.2.2	The rationale for the dose of methadone if outside the therapeutic range is recorded	171	168 (98.2)
3.3.1^a^	Patients have frequency of supervised dispensing recorded	182	178 (97.8)
3.3.2	Patients have rationale for supervised dispensing recorded	182	178 (97.8)
3.4.1	Is there a record of a minimum of one randomised drug screen per month?	180	163 (90.5)
3.4.2	All drug screens are positive for methadone/EDDP	177	172 (97.2)
3.5.1^a^	There is a record if patients are using other substances along with their methadone	158	156 (98.7)
3.5.2^a^	There is evidence of the interventions made if patients are using other substances along with their methadone (opiates, benzodiazepines, cocaine, alcohol, cannabis etc.)	144	117 (81.2)
3.6.1^a^	All patients who are prescribed Benzodiazepines should have the rationale for prescribing recorded in their notes	133	122 (91.7)
3.6.2^a^	All patients who are prescribed Benzodiazepines should have a review of the prescription in the past 3 months recorded in their notes	133	117(88)
3.6.3^a^	All patients who are prescribed Benzodiazepines should have the dose recorded in their notes	133	132 (99.2)
3.6.4^a^	All patients who are prescribed Benzodiazepines should have evidence of 3 monthly reviews recorded in their notes	120	120(100)
3.6.5^a^	All patients who are prescribed Benzodiazepines should have evidence of communication with prescriber (if GP is not main prescriber) recorded in their notes	51	29(56.9)
3.7.1^a^	All patients who are prescribed Z-Hypnotics should have the rationale for prescribing recorded in their notes	93	89 (95.7)
3.7.2^a^	All patients who are prescribed Z-Hypnotics should have a review of the prescription in the last 3 months recorded in their notes	94	84 (89.3)
3.7.3^a^	All patients who are prescribed Z-Hypnotics should have the dose recorded in their notes	91	91 (100)
3.7.4^a^	All patients who are prescribed Z-Hypnotics should have evidence of 3 monthly reviews recorded in their notes	82	82 (100)
3.7.5^a^	All patients who are prescribed Z-Hypnotics should have evidence of communication with prescriber (if GP is not main prescriber) recorded in their notes	14	10 (71.4)
4.1.1^a^	There is a record that patients at risk of blood borne viruses have been offered screening for HIV	182	155(85.2)
4.1.2^a^	There is a record that patients at risk of blood borne viruses have been offered screening for Hepatitis C	182	154(84.6)
4.1.3^a^	There is a record that patients at risk of blood borne viruses have been offered screening for Hepatitis B	182	150(82.4)
4.1.4	There is a record that patients at risk of blood borne viruses have been offered screening for Hepatitis A	181	80(44.2)
4.2.1^a^	There is evidence that patients who are HIV positive have been referred to specialist service.	38	37(97.4)
4.2.2^a^	There is evidence that patients who are Hepatitis C antibody positive and PCR positive/ PCR testing not available have been referred to specialist service as appropriate.	127	122(96.1)
4.3.1^a^	There is evidence that patients who are Hep B negative have been offered vaccination.	172	115(66.9)
4.3.2	All patients who have completed Hep B vaccination regime have been offered post vaccination Hep B antibody test	145	81(55.9)
4.3.3	There is evidence that patients who are Hep A antibody negative have been offered Hep A vaccination	138	60(43.5)

^a^denotes essential criteria

### Phase three evaluation of GP experience (qualitative)

Three themes emerged from the content analysis; *‘Reflective and Informed Practice’; ‘MTP Patient Care’* and *‘Recommendations to Improve the Online Audit’.* Each theme is presented with descriptive data and illustrative quotes.

#### Theme one: Reflective and informed practice

Participants generally found the online MTP self-audit easy to complete (see Table 4), with 92.86% reporting that the tool was helpful in reflection on their clinical practice.

**Table 4:** How did you find the MTP self-audit survey to complete?

How did you find the MTP self audit survey to complete?	Number of Participants n(%)
Very Easy	16 (28.6)
Easy	26 (46.4)
Neutral	9(16.1)
Difficult	4 (7.1)
Very Difficult	1 (1.8)

44 participants described how the online MTP self-audit assisted their reflective practice, and reminded them of the importance of standards and parameters of good patient care. It reminded them of key protocols of care and deficiencies warranting attention, and overall contributed to more comprehensive care, documentation and improving processes, standards and management using a systematic approach.

*‘It allowed me to reflect on the type of care I was providing to my patients on MMT. Often when managing chronic illnesses and treating patients that you see regularly you miss the wood form the trees. It also allowed me to see how I was documenting the care I was providing’. Participant #38.*

*‘It is always useful to go through notes in an analytical way - it reinforces standards needed and is a useful way of ensuring we are meeting targets.’ Participant #39* ***.***

Completion of the online MTP self-audit improved practice and compliance to the MTP in 67.92% of participants. Participant practice improvements centred on accountability in using closer and improved review and data systems (doctor, *Excel* and software), note and record keeping, and patient informed consent. Some participants described using available software tools, and creating checklists on their practice systems so as to monitor their care of methadone patients according to the audit criteria, and to assist them in follow up online audit completion. The online self-audit was viewed as an excellent **‘***reminder’* on aspects of patient care, ‘*reinforces the guidelines for best practice’* and a way of ‘*organising information in an accessible manner’.*

*‘It alerted me to areas of my work that needed attention.’ Participant #4.*

*‘Identify blind spots and room for improvement.’ Participant #5.*

#### Theme two: MTP patient care

62.69% of participants were of the view that the audit would improve patient care. This was commented to be underpinned by an improved rapport with the doctor, and ultimately improve patient health outcomes (*‘It will identify gaps in quality of care’*). Particular areas relating to the MTP standards and provision of MMT centred on closer adherence to clinical guidelines for opiate substitution treatment (OST), patient safety, awareness of related health problems, need for regular urine screening, attention to benzodiazepine and other psychoactive drug prescribing and monitoring of patient dependence, serology (in particular Hepatitis A and B screening, vaccination and titre status, and referral to specialist services), documentation of patient doctor discussions around illicit psychoactive drug use, and the necessity to accurately document all aspects of patient consultations around the key MTP audit criteria.

*‘Consideration of all drug misuse rather than focusing on opiates which can happen. It prompted me to review all my patients’ serology. It forced me to look at the patients initial assessment forms and to complete some information that had been missing after transfer but that I had omitted to get until the time of the audit.’ Participant #13.*

*‘It highlighted that records for things such as transfer information, hepatitis B titres and other information is lacking in the charts. This is because many of our patients have been on methadone therapy for over twenty years.’ Participant #17.*

Completing the online self-audit gave participants confidence, upskilled participants in line with current evidence base, and cemented the desire for some to continue their work as Level 1 providers (for stabilised patients in the community) and to become specialist trained in initiating MMT in addiction clinics (ICGP Level 2). Others commented on their intention to use the online self-audit regularly to monitor their progress.

#### Theme three: Recommendations to improve the online audit

55.77% of evaluation participants had previously had a paper based external audit where the clinical audit facilitator at the ICGP visited the practice and conducted the audit. The majority found the online audit better in comparison. See Table 5.

*‘I felt more involved than with the previous audit done by someone else.’ Participant #20.*

**Table 5:** How did the online audit compare with the paper based external audit?

How did the online audit compare with the paper based external audit?	Responses (%)
Much Better	24.1
Better	48.3
No Different	24.1
Dis-improvement	3.5

The majority of evaluation participants were very positive around the online audit experience. One participant stated *‘audits are a waste of time.’*

*‘Superb audit. Online format excellent. Very impressive and it showed gaps, lacks in my practice. Probably the first worthwhile audit I have done.’ Participant #10.*

*The tool was easy to use, response from the college was fast and efficient. It was great to have an electronic copy of my audit to upload to my CPD folder - I also think that as a tool it could have great utility as be used for the management of other chronic illnesses.’ Participant #19.*

One participant was negative in their view of the online audit and said.

*In my opinion “fail” criteria need to be modified to reflect things that can actually be remedied - I was failed mainly on the basis of a misunderstanding of the hep b questions and also on a lack of old transfer documents which preceded my care of the patients - this suggests that the audit tool is being used on its own to decide pass/ fail and that it is not being looked at in context. I have found the whole experience negative and frustrating and still have to repeat the whole audit again without having really been able to remedy the problems.’ Participant #8.*

Some participants found the online audit lengthy and protracted. Another commented on the need to monitor a smaller number of cases over a longer timeframe, for example 12 months.

***‘*** *Less is more. The survey was onerous- much of the data gathering was pointless. Furthermore I felt it treated me like I was a monkey rather than a trained medical practitioner. Participant #5.*

Recommendations on methods to improve the online audit centred on improving the clarity of some questions (for example hepatitis titres) ‘*(‘some of the questions need improved clarity - some slightly ambiguous’)*, eliminating repetition, reducing the emphasis on historial information, and including all possible options and patient rotation within practices.

*‘Questions on hepatitis titres very unclear. Also too much emphasis on historical information which can’t be changed.’ Participant #2.*

*‘Some questions were black & white where there was another option. For example, did patient have blood test yes or refusal. In the patients case he did not refuse, took the blood form but did not attend for blood test.’ Participant #13.*

*‘Need to accommodate group practice better- i.e. patients rotate amongst DOCs for different but often very valid reasons.’ Participant #23.*

*‘It needs to look at linear profile- ie a smaller number of patients over a longer period- eg a year.’ Participant #6.*

Some evaluation participants commented on the need for a useful checklist of items.

*‘A checklist of items to be audited would be very helpful when extracting data from records, would be easier than hopping back and forth between website and records’. Participant #4.*

One participant commented on the restriction of the online tool and requested additional space to provide open ended answers to support rationales for treatment and patient care.

*I think, it is largely a box ticking exercise. There should be qualitative information provided which would explain rationale better. Participant #4.*

Difficulties were observed with regard to the standard of communication with other prescribers of benzodiazepines and Z-drugs, due to issues of patient confidentiality. Some observed the need for provision of written benzodiazepine prescribing guidelines to incorporate into their practice.

*Regarding contact with benzodiazepine prescribing doctors - as some patients are not mine, this may prove difficult - due to patient confidentiality issues* etc.*’ Participant #14.*

## Discussion

We present here the successes and recommendations for improved MTP audit cycling in the Irish MMT system. General practice settings are potent learning environments [23] and the MTP audit outcomes are positive in terms of encouraging reflective and evidence based informed practice, and ultimately improve safety in methadone prescribing, patient care and the management of associated health conditions. The response rate of 53% (182/345) was viewed by the team as a good response and reflects a much higher response that has been achieved with previous Irish on-line studies. A reduced number (132) completed the final audit representing 38% of the GPs providing MMT in Ireland. It is likely that this may represent a sampling bias, with more enthusiastic, better organised and more complaint GPs engaging in the audit process. The added benefit of adapting this structured approach to audit is that it also identifies those not engaging in the process and allows the ICGP to target this group with more support to encourage them to engage. The ICGP plans to undertake further research on this group to identify barriers to their involvement in the MMT audit. This may inform how the process is developed going forward. It is worth noting that this is the first full year of this particular audit cycle and the ICGP plan to develop more targeted audits focusing on different aspects of MMT management using the same platform and structures.

To our knowledge this is the first and only on-line national audit structure that monitors the care provided by GPs for a chronic medical condition. While not yet providing full coverage for all GPs providing MMT in Ireland it is a good start and has the capacity and utility to expand. The linking of this audit with the National Irish Medical Council (IMC) Audit should encourage more GPs to engage in the future. This model also provides a template for the ICGP to develop further on-line audits as educational and monitoring tool to monitor the care provided for other chronic medical conditions such as diabetes and hypertension. While requiring further evaluation and modification we believe that this novel approach to audit can be adapted for use in other jurisdictions in both clinical practise and under and post graduate training. The matching of the audit criteria with the national standards for the treatment of opioid dependence additionally provides a mechanism to disseminate updated standards to practising GPs and also a means to monitor the levels of compliance as patterns and trends in opioid dependence change in Ireland. It may also potentially incur have the benefit of Irish GPs standardising care around such standards /guidelines, which ultimately may improve and simplify the management and care of complex opioid dependent patients.

The blended approach of online self and external audit, with evaluation has presented the ICGP with a collective insight into the complexities of learning in the MMT practice, and how audit processes can improve practice functioning, patient care and further inform the MTP audit process [16, 24–27]. The online MTP self-audit was well received and encouraged reflective practice in GPs providing MMT in Ireland. The process hinged on effective individualised, educational feedback and learning support provided by the clinical audit facilitator via email and in person during paper based audits at concurrent points in the audit cycle, and the individual GP’s ability to review and critically analyse their professional practice, and manage change. The MTP audit given its situation within the ICGP as national CPD provider and situated experiential learning process, will further support attainment of knowledge and learning embedded within social connectivity in primary practice [28–31]. We recognise that workplace learning is situated within complex environments [16, 32], and most particularly so given the vulnerabilities and complexities in treating and caring for opioid dependent patients in primary care. The audit process supports the evidence base which posits that learning outcomes are supported by socio cultural, cognitive and reflective views on practice based learning [16, 33, 34], triggered by real life practice based experiences [31, 35–37] and situated within the individual GPs cultural and localised context within their own developmental space [16]. This is particularly applicable to the concept of treating and caring for complex patients with opioid dependence in primary care.

The ICGP as national training provider for GPs, and facilitator of the MTP audit process is cognisant of the need to stimulate and encourage provision of methadone treatment provision within primary care in Ireland. At a micro level, not all GPs wish to be trained in the treatment and care of drug users, or provide opioid agonist treatment, or wish to have methadone patients in the practice. Stigma and negative attitudes toward opiate dependence and substitution treatment remain an issue on both training uptake, and engagement with the MTP, and impacts on the degree to which methadone treatment is provided in practice. The ICGP’s auditing approach whilst focusing on the cognitive dimensions of knowledge acquisition, reflective practice and modelling, also considers the dimensions of socio cultural environments, particularly in the form of favourable work environments, strong individual goals in attaining the MTP standards of care and aspirations in becoming excellent Addiction Medicine professionals, the structuring of experience as opposed to dissemination of knowledge, and the grounding of the MTP audit within a community of trained GPs. Such learning processes within workplace pedagogy [38] are underpinned by this audit and shaped at micro level by the GPs identity as methadone prescriber, knowledge acquisition, opportunities for practice engagement and social membership within the realm of Addiction Medicine. Audit innovations such as this one developed and evaluated by the ICGP are increasingly centred on ownership and internal resolve [39], with motivational change and mechanisms for change understood within the dynamics of knowledge, attitude and behaviour [40], all of which are socially and culturally mediated. Multiple actors were evident in this novel audit process conducted for the first time in Ireland in the form of methadone patients themselves, practice nurses, practice managers, peers, the clinical audit facilitator, the ICGP staff, social workers and trained GPs. The feedback style of the clinical audit facilitator, timely of provision of feedback and paper based audits have the potential to enhance feelings of competence and confidence, and further support the GP within their own practice as personal learning environment [32, 41, 42]. Feedback is most effective when baseline performance is low, when behavioural change is provider driver and when learner GPs are targeted [39]. Whilst the MTP online self-audit is essentially an individualised exercise, with interaction, feedback and support from the clinical audit facilitator, the community itself at the SMP of the ICGP provides continued support in the form of regular webinars where GPs can dial in and discuss complex cases, mentoring and work with other more experienced GPs, and placements in clinics for Level 1 GPs seeking further training experience and the opportunity to work with patients commencing methadone treatment. These built in aspects of the MTP audit facilitate supported and participatory learning whilst participating in the audit process with colleagues [29, 43].

Of note was the majority positive experience with the MTP audit process, revealing a commitment to continuing to provide MMT and upskill to specialised Level 2 practitioners. Ideal scenarios underpinned by *‘deep learning’* consist of Level 1 GPs mentored by more experienced GPs in the practice or locality (see Fuller & Unwin, [44]). Using the socio cultural framework approach, professional discourse around opiate dependence, provision of methadone treatment in primary care practices, and sharing of experiences can redefine professional identities in Addiction Medicine and attitudes towards methadone treatment itself, assimilate GPs into the MTP system and transform social practice and communities of learners [1, 45]. Transitioning from Level 1 GP responsible for stabilised methadone patients in the primary care community setting, to that of the more Specialised Level 2 GP who initiates methadone treatment and stabilises the patient, represent an interesting parallel described by Lave and Wenger [45] from that of newcomer or novice to that of old timer or mentor in *“legitimate peripheral participation in communities of practice”*, with newcomers steadily moving toward their place as core of the community (in this instance Addiction Medicine community).

## Conclusions

The paper essentially documents and describes a design, implementation and evaluation process for a novel audit targeting methadone treatment in Ireland, and is intended to be a useful publication for general practice improvement, and with a specific focus on general practice providing care for complex patients with opioid dependence. Results from this novel audit process demonstrate a high level of compliance with best practice MTP guidelines by Irish GPs. The process hinged on the individual GP’s ability to review and critically analyse their professional practice, and manage change. The online self-audit process was generally well received, with some suggestions for improvement, and encouraged reflective practice. In terms of understanding the complexities of improving treatment and care of those with opioid dependence in primary care, we additionally recognise that GP training in the delivery and management of MMT in primary care can be better understood using a socio-cultural perspective to clarify and understand learning processes, appreciate their developmental space, develop their professional identities as methadone doctors, and understand perceived stigma of addiction. Ultimately GP education and support using innovative audit cycles, can create a conceptual framework for understanding how experiential work contexts, patient and professional connectivity, an appreciation of the psycho-social aspects of drug addiction, and the development of positive emotions/attitudes and therapeutic alliances with their methadone patients.

## Abbreviations

ARG: Audit Review Group
BBVs: Blood Borne Viruses
CPD: Continuing Professional Development
GPs: General Practitioners
ICGP: Irish College of General Practitioners
IMC: Irish Medical Council
MMT: Methadone Maintenance Treatment
MTP: Methadone Treatment Protocol
OST: Opiate Substitution Treatment
SMP: Substance Misuse Programme
